# Supplementary figures and images for: Dynamic bandwidth allocation in time division multiplexed passive optical networks: a dual-standard analysis of ITU-T and IEEE standard algorithms
Source: PeerJ Comput Sci. 2025 May 9;11:e2863. doi: 10.7717/peerj-cs.2863 (PMC12192646; doi:10.7717/peerj-cs.2863)

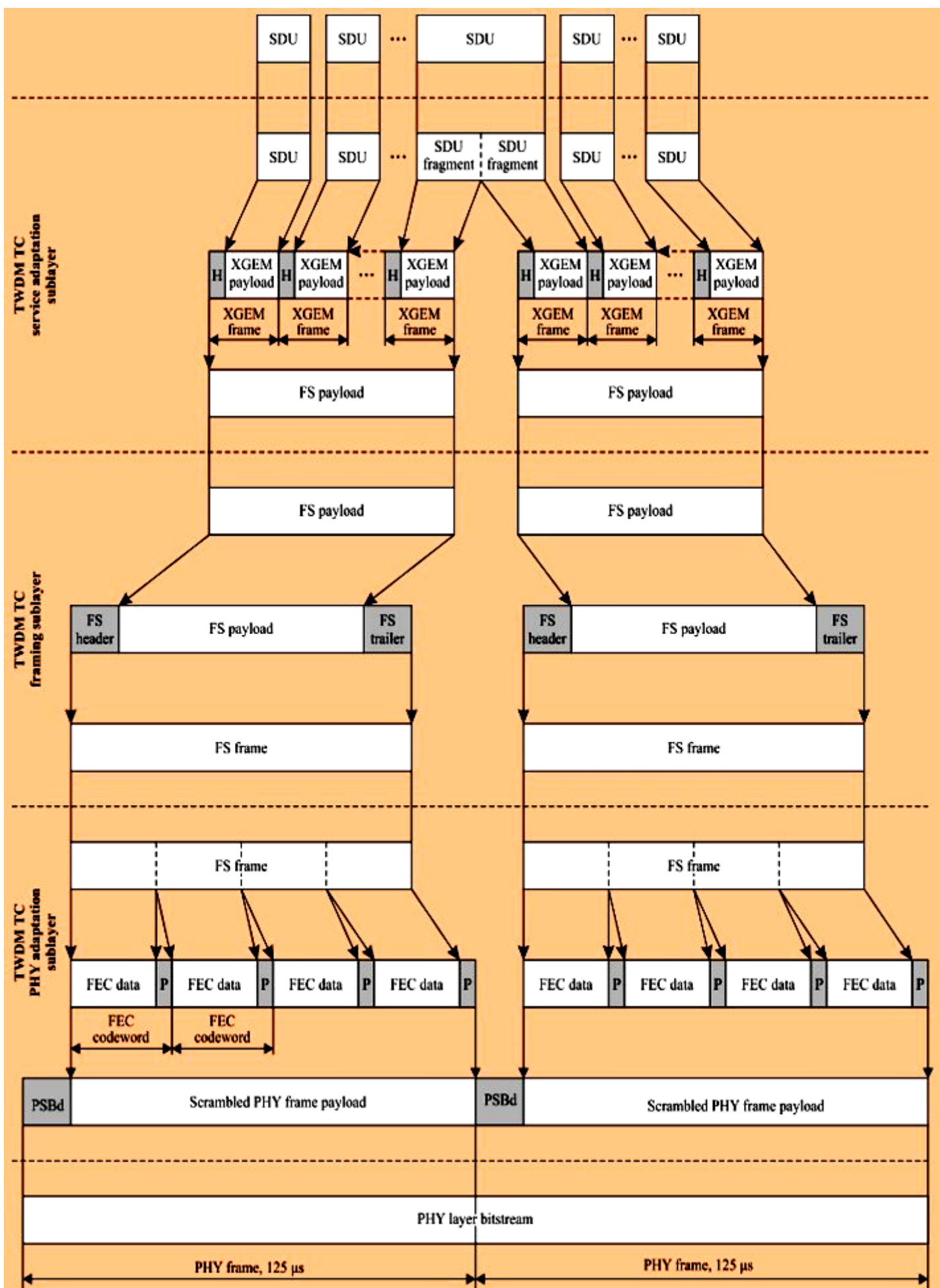

H – XGEM frame header

P – FEC parity

NOTE – FEC encoding of an FS frame is a static run-time option

Supplement: Supplemental Information 1 [file peerj-cs-11-2863-s001.pdf]
