# Supplementary material for: Dynamic bandwidth allocation in time division multiplexed passive optical networks: a dual-standard analysis of ITU-T and IEEE standard algorithms
Source: PeerJ Comput Sci. 2025 May 9;11:e2863. doi: 10.7717/peerj-cs.2863 (PMC12192646; doi:10.7717/peerj-cs.2863)
Supplement: Supplemental Information 2 [file peerj-cs-11-2863-s002.pdf]

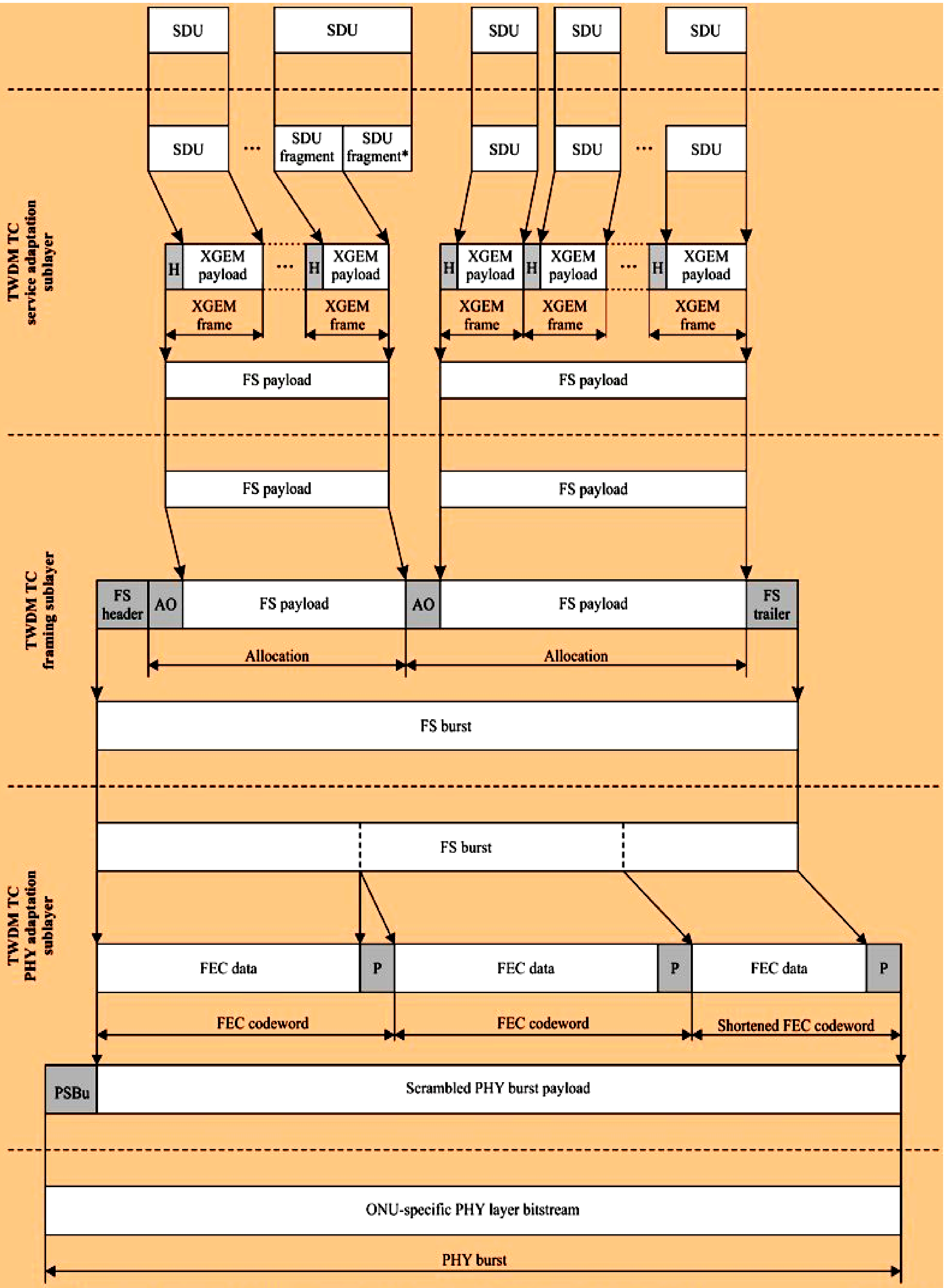

(*) The remaining fragment of the SDU is transmitted in the subsequent allocation with the same Alloc-ID.
H – XGEM frame header
AO – Allocation overhead
P – FEC parity
